# Allocating Logging Rights in Peruvian Amazonia—Does It Matter to Be Local?

**DOI:** 10.1371/journal.pone.0019704

**Published:** 2011-05-11

**Authors:** Matti Salo, Samuli Helle, Tuuli Toivonen

**Affiliations:** 1 Department of Biology, University of Turku, Turku, Finland; 2 Department of Geosciences and Geography, University of Helsinki, Helsinki, Finland; Texas A&M University, United States of America

## Abstract

**Background:**

The fate of tropical forests is a global concern, yet many far-reaching decisions affecting forest resources are made locally. We explore allocation of logging rights using a case study from Loreto, Peruvian Amazonia, where millions of hectares of tropical rainforest were offered for concession in a competitive tendering process that addressed issues related to locality.

**Methodology/Principal Findings:**

After briefly presenting the study area and the tendering process, we identify and define local and non-local actors taking part in the concession process. We then analyse their tenders, results of the tendering, and attributes of the concession areas. Our results show that there was more offer than demand for concession land in the tendering. The number of tenders the concession areas received was related to their size and geographic location in relation to the major cities, but not to their estimated timber volumes or median distances from transport routes. Small and Loreto-based actors offered lower yearly area-based fees compared to larger ones, but the offers did not significantly affect the results of the tenders. Local experience in the form of logging history or residence near the solicited concession areas, as well as being registered in the region of Loreto, improved the success of the tenders.

**Conclusions/Significance:**

The allocation process left a considerable number of forest areas under the management of small and local actors, and if Peru is to reach its goal of zero deforestation rate by safeguarding 75 per cent of its forests by 2020, the small and the local actors need to be integrated to the forest regime as important constituents of its legitimacy.

## Introduction

The fate of tropical forests is a global environmental issue [1], yet many far-reaching decisions concerning forest resources are made at local level [2]. Peru possesses the fourth largest tropical rainforests on Earth. The country has recently set the ambitious goals of safeguarding 75 per cent of its forests, and reducing deforestation rate to zero by 2020, through the “National Programme of Forest Conservation for Climate Change Mitigation” [3]. However, the combination of extensive forests, sparsely distributed valuable timber, difficult physical access, high level of poverty, widespread unemployment, and insufficient funding for control and monitoring have made it difficult for Peruvian authorities to efficiently enforce formal rules regulating access, logging activities, and forest management [4]–[6].

In Peru, all natural resources, including forests, are owned by the state as a part of the national wealth. Up until the early 2000s, the Peruvian access regime to forest resources was in effect based on, and ideally suited for, migratory selective harvest; logging permits were short in duration and small in extent [7]. A new forest law passed in 2000 [8] and implemented during the 2000s pursued to change this habit by e.g. introducing long term forest concessions as the main access mechanism to forests [5], [9]. Since then, however, the Peruvian forest regime has been in a constant turmoil; new reforms have been implemented and new forest values, such as ecosystem services, introduced [10]. Renegotiation of power-relations between the state, private, and communal actors has also led to major protests against regime changes perceived unjust by local communities [4]–[5], [9], [11]. We argue that lack of locally perceived legitimacy is one of the most important underlying reasons for the troublesome implementation of the recent forest sector reforms in Peru.

In any case, more than 7 million hectares of forest concessions have to date been allocated in separate competitive tenderings in different Amazonian regions of Peru [5], [9], and they are likely to remain a central part of any future access regime. Forest concessions are formal contracts between forest owner (in the Peruvian case this is the state) and another party (concession holder, concessionaire), by which the concession holder leases a right to exploit forest resources, accompanied by the obligation to manage them according to legally established principles and methods within a specified area and a specified time-frame [12]. The success or failure of the Peruvian concessions potentially has substantial long-term effects on forest disturbance rates and biological diversity in the region [13], considering that the concession period is 40 years, which, in the moment of expiration, can be renewed by the parties [8].

In this study, we examine the allocation process of logging rights through forest concessions in Loreto, Peruvian Amazonia, where more than 4 million hectares of tropical lowland rainforest were offered for tender in a competitive allocation process (tendering) in the year 2004. We first briefly describe the study area and the concession allocation process in Loreto in 2004. In continuation, we analyse a number of data sets describing the concession areas (hereafter concession units), as well as actors that took part in the tendering, their tenders, and the results of the process. In order to explore the role of locality in the allocation process, we test which attributes of the concession units were related to the number of tenders they received, and then assess the differences between local and non-local actors regarding their economic offers and their success in the tendering. Finally, we discuss the implications of locality on the current and future forest governance and its legitimacy in Amazonia.

## Materials and Methods

### Study area and allocation process

**Figure 1:**
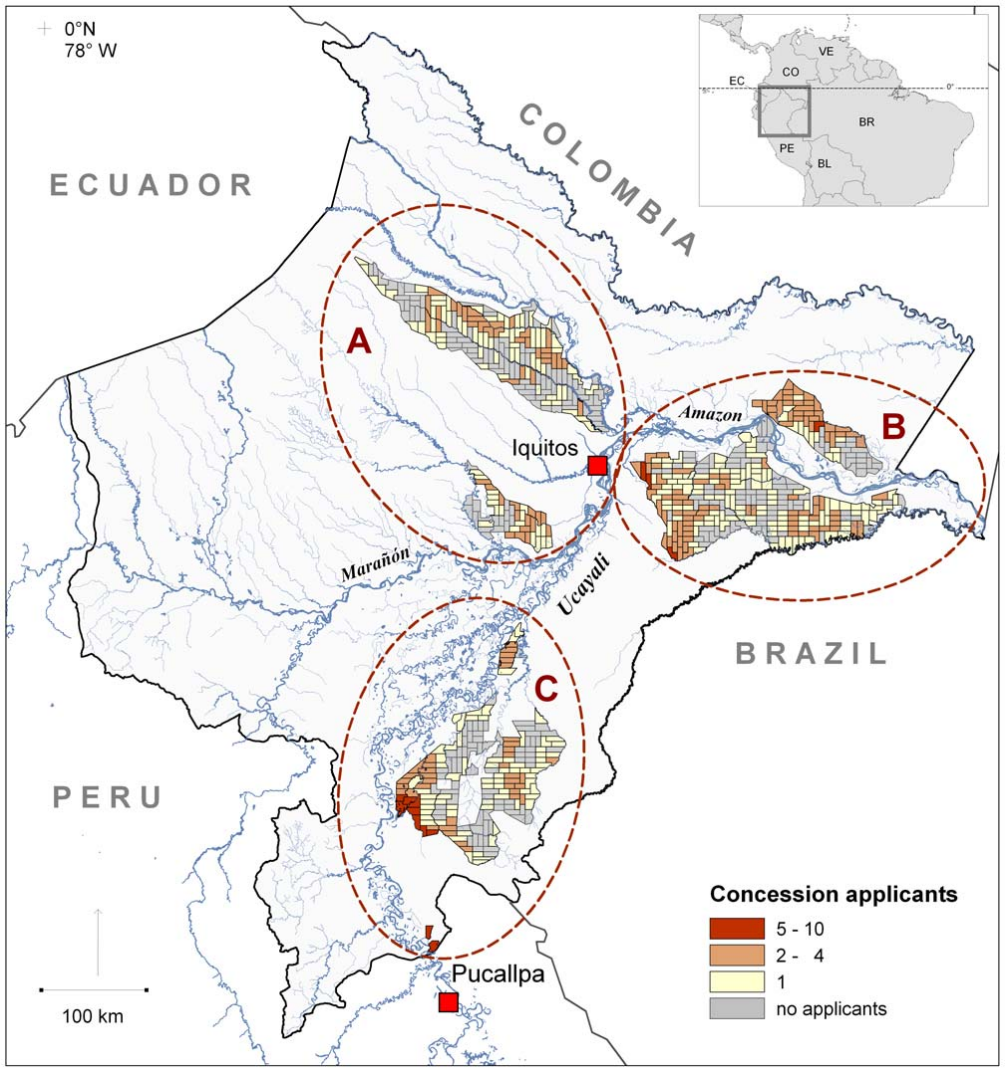
Forest concession units in Loreto, and the number of tenders (concession dapplicants) they received in the allocation process 2004. The cities of Iquitos and Pucallpa are marked with red squares. The concession Blocks are marked with dashed-line ovals indicated with letters A, B, and C.

Loreto is the largest of the 25 Peruvian regions with an extension of 368,900 km^2^, equalling the size of Germany, and comprising more than 28 percent of the Peruvian territory (Figure 1). The tropical lowland rainforests covering the area are among the most diverse ecosystems on Earth [14], and harbour a particularly high diversity of trees [15]. Despite its size, Loreto's human population is less than 1 million (<4 percent of all Peruvians), of which c. 500,000 live in and around the region's capital city Iquitos. Loreto is geographically and culturally relatively isolated and has a strong regional identity [16]. It also has a tradition of export-led forestry [17]. Although long distances and the almost complete lack of terrestrial roads form a constant challenge, forestry is one of the most important economic activities in the region. According to Tello Fernández et al. [17], forestry contributes to around 50 per cent of rural jobs, and forms more than 70 per cent of the value of all exports in Loreto.

In Loreto the forest areas eligible for concession were delimited in 2001 [9]. The tendering was opened in June 2002, and suspended the next month [18] mainly due to strong criticism from local timber companies [5]. The companies' concern was based on at least two obvious reasons: first, new competitors would potentially either enter, or emerge within, the region; and second, the cost of forest management and timber extraction would potentially increase, due to new legal requirements. There was no legal restriction for foreign participation in the concession process, however the tenderings' scoring system, favouring residence or former logging contracts near the solicited concession areas, in addition to tight schedules, hindered international participation. This subtle exclusion of foreigners and the consequent inclusion of the largely informal local small-scale extractors into the formal access-regime was apparently one way to legitimise the reform.

The tendering was reopened in November 2003. Overall, 749 concession units with a total area of 4,644,163 hectares, were offered, the average size of a concession unit being 6,200 hectares (min = 5,000 ha, max = 9,944 ha). The base documents of the tendering were available at a cost of 30 Peruvian nuevos soles (c. 8.50 USD), and a total of 726 base documents were sold. Each applicant submitted a technical tender and an economic offer. The technical tender included information on the applicant's experience and locality, available assets, and work plan, with a maximum score of 100 points. An important feature of the scoring system was that it explicitly favoured local actors; the closer to the solicited concession unit the applicant resided or had past logging contracts, the higher the score obtained.

The economic offer, in turn, was the yearly area-based fee the applicant promised to pay for the total of the concession area. The score of the economic offer was calculated by dividing each offer by the highest bid in the same unit and multiplying the quotient by 100; the maximum score thus being 100. The base value (starting price) of the area-based concession fee was 0.40 USD/ha/year [19], which was uniform in all of the concession units nationwide regardless of their location or production potential.

In order to weight the technical tender in comparison to the economic offer, the technical tender was valued as having a weight of 90 per cent of the total score of the applicant, while the economic offer was worth 10 per cent. This decision was made in order to avoid the tendering turning into an auction, which would arguably discriminate against local actors with limited capital assets. An auction could also potentially form an incentive for unrealistically high offers that had been seen in former allocation processes in Madre de Dios and Ucayali regions, where the first tenderings gave more weight to the economic offer [20].

The total number of actors taking part in the tendering was 328, and 64 per cent (n = 211) of them won a concession. The most numerous group of the participants were individuals (70 per cent, n = 230), whereas companies constituted a quarter of the applicants (25 per cent, n = 82), the remainder being partnerships (associations of two or more individuals committed to form a formal association or company in case of winning a concession; 5 per cent, n = 15) and one Non-Governmental Organisation (NGO). The tendering was concluded in May 2004, and the vast majority, 98 per cent (n = 206), proceeded to sign a concession contract within the next year.

### Data and variables used in the analysis of the tendering

We first classified the actors' locality in order to test differences between locals' and outsiders' behaviour and success in the tendering. The distinction between locals and outsiders is common in literature, but as a concept it is often troublesome and vaguely elaborated [21]. We used a combination of two different criteria derived from separate sources. First, we made a distinction between actors that were registered in Loreto and the ones that were registered outside the region; and second, we classified the same actors in two groups according to their direct experience related to the surroundings of the concession units that they solicited. For the analysis, we cross-classified the applicants taking part in the tendering into four locality classes as presented in Table 1.

**Table 1:** Classification of the locality of the applicants based on INRENA tendering data and SUNAT database [22].

	*SUNAT ‘Loretans’*	*SUNAT ‘outsiders’*
**INRENA ‘locals’**	local Loretans	local outsiders
**INRENA ‘non-locals’**	non-local Loretans	non-local outsiders

For the first classification, we used the internet database of the Peruvian taxation authorities, SUNAT (Superintendencia Nacional de Administración Tributaria) [22]. We retrieved the data using the name of the applicant (individual, company, or partnership) as a key word, and as a result we created a data set with the registered office or domicile of all formalised economic actors taking part in the tendering. All applicants with an office or domicile registered by SUNAT in the region of Loreto were labelled ‘Loretans’ and the ones registered in any other region or outside Peru were labelled ‘outsiders’.

The second classification was based on the tendering data provided by INRENA (Instituto Nacional de Recursos Naturales), an institution under the Ministry of Agriculture and at the time of the tendering directly responsible for the management of renewable natural resources. In our classification all participants either residing or with past logging contracts within the watershed where they solicited a concession unit were labelled ‘locals’, whereas all the rest were labelled ‘non-locals’. We refer to “watersheds” as a category used by the forest authorities in order to classify the applicants. We chose the watershed level, as used by INRENA in the tendering scoring system, to be the appropriate spatial resolution for this classification because Loreto lacks a large-scale terrestrial road network and practically all timber logged is transported fluvially. Thus the mouths of the rivers are currently the most feasible, if not the only, locations for systematically controlling the transportation of logged timber.

In the analysis, we first wanted to explore the general popularity of the different concession units by measuring the concession popularity as the number of tenders the units received. In order to study the effects of the geographical location of the units, we attached six attributes to the concession units: concession Block, surface area (in hectares), skidding distance (km), distance to closest city (km), closest city (Iquitos or Pucallpa), and estimated timber volume (m^3^) (Table 2). The division of concession Blocks is shown in Figure 1. Skidding distance describes the median Euclidean distance to the nearest river, which we use as a proxy for the cost of transporting felled logs to the primary transport routes formed by rivers. The skidding distance was defined based on a river data set manually digitised from 30-meter resolution Landsat TM imagery by a Finnish-Peruvian environmental cooperation project, Biodamaz [23]. Although river access depends ultimately on water levels in tributary waterways, of which many are too small to be included in our data, our analysis is more realistic and detailed than the analyses using the distance as the crow flies as a proxy for accessibility. The same river network data was used to measure the distances from the concession units to the main timber trade centres, the cities of Iquitos and Pucallpa. The distance from both cities along the river network were calculated using the cost-distance function of ArcGIS 9.2, and the resulting distance to the closest of these cities was stored as the variable ‘distance to closest city’. In addition, the variable ‘closest city’ had a value of either Iquitos or Pucallpa, depending on which of these two cities was closer, via river, to the concession unit in question.

**Table 2:** Attributes of the applicants and the concession units used as explanatory variables in the models.

	*Attribute (explanatory variable)*	*Classes/units*
**Applicants**	*locality*	*local Loretans, non-local Loretans, local outsiders, non-local outsiders*
	*scale*	*small extractors, medium-sized actors*
	*type*	*individuals, companies, partnerships*
	*area-based fee*	*USD/ha/year*
**Concession units**	*concession Block*	*A, B, C (see * *Figure 1* *)*
	*concession unit area*	≤6004 ha, >6004* ha (*median)
	*skidding distance*	≤6.65 km, >6.65* km (*median)
	*distance to closest city*	*<500 km, 500–700 km, >700 km*
	*closest city*	*Iquitos, Pucallpa*
	*estimated timber volume/ha*	*<80 m^3^, 80 m^3^*

The base documents of the tendering provided a description of forest types found within the concession units, and the area these types covered in each of the units. This information was based on inventories carried out by INRENA [24] and contained volumetric estimations of timber resources technically available in different forest types. The data provided theoretical upper and lower limits for timber volumes, and we used the lower figures to calculate an estimate of minimum timber volume per hectare technically available for each concession unit. We acknowledge limitations in the quality of this data set due to the preliminary nature of its analysis and inaccurate input data used. However, we included the variable ‘estimated timber volume’ in our models because the data was publicly available for all the applicants, and thus potentially influenced their decisions.

We were also interested in the variables related to the applicants' economic offers, and to their probabilities of winning a concession. Thus, we selected the yearly area-based fee offered and the result of the tender as response variables in our analyses. We decided to study the economic offer, expressed as an area-based fee per year, because it reflects the actor's willingness to pay for the concession rights in the long term. The actor's success, expressed as the probability of winning a concession, was studied because it can be used to assess the tendering system's potential bias towards local actors. ‘Area-based fee’ was also used as an explanatory variable when the probability of winning was modelled. The variables stored as attributes of the applicants and the concession units are presented in Table 2.

Due to the type and the scale of the actors taking part in the tendering being potentially related to the economic offers they make, we classified the applicants according to scale and type. The participants could either solicit only one concession unit, or more than one. In the former case the applicant belonged to the scale class ‘small extractors’ and in the latter to the class ‘medium-sized actors’. In the tendering scoring system, small extractors were favoured by higher scores. Furthermore, the variable ‘type of actor’ was classified into three categories: ‘individual’, ‘company’, and ‘partnership’. The economic offer of the applicant in USD/ha/year was stored as variable ‘area-based fee’. Moreover, in addition to the attributes of the applicants, we wanted to capture the possible effects of the geographic location, size, and accessibility of the concession units on the economic offers the participants made, and therefore we used the variables based on the concession units' attributes described above.

### Statistical analyses

In order to study the behaviour of the different actors taking part in the tendering, we examined whether the explanatory variables (Table 2) were associated with the number of tenders per concession unit, the area-based fees offered by the applicants, and the applicants' probability in winning the solicited concession unit. The associations between the explanatory variables and the number of tenders the concession units received, and the area-based fee offered by the applicants, were examined with regression models assuming Poisson distributed errors and log link function, since these responses were count variables. The influence of the explanatory variables on whether the applicant won the concession unit or not was examined using logistic regression model, with binomial errors and logit link function. When needed, Pearson's χ^2^ was used to rescale the parameter covariance matrix to adjust for any under- or overdispersion.

Prior to the analyses, the variables of ‘concession unit area’, ‘median skidding distance’, and ‘estimated timber volume’ were divided in two categories, based on the variables' median values (to distinguish between large and small, close and remote, and abundant and scarce units). Meanwhile, the variable ‘distance to the closest city’ was divided in three classes, to detect the possible effect of remoteness (implying less extraction pressure and thus more available timber on the one hand, and more difficult access on the other, which we hypothesised could favour intermediate distances). The breaking points of these classes are presented in the Table 2. All concession units were included in the analysis of popularity, but only the units that received tenders were included in the analyses of economic offers and tendering success. To account for the facts that some applicants made a tender for several concessions, and several concession units had multiple applicants, we applied Generalized Estimating Equations (GEE) to account for the respective correlation structure in the models for the area-based fees offered by the applicants, and for the probability of the applicants for winning the solicited concession unit [25]. Unstructured working correlation matrix, with concession identity nested within applicant identity, was used to accomodate these correlations [25]. Statistical inference was based on Score test [25]. No stepwise model reduction was applied because such methods dramatically increase the rate of type I errors [26], and because our aim here was to obtain the most accurate point estimates and their confidence intervals (CI) [27]. In the case of statistically significant association between categorical variables having more than two levels and the response, statistical interpretation of the group-differences was based on the 95% confidence intervals of the means. For example, if the 95% confidence intervals of means overlap half the length of one arm, this corresponds approximately to statistical significance at p = 0.05 [28]. All analyses were conducted with SAS statistical software version 9.2 (SAS Institute Inc, Cary, North Carolina, USA).

## Results

### Demand and offer of forest land: popularity of concession units

**Figure 2:**
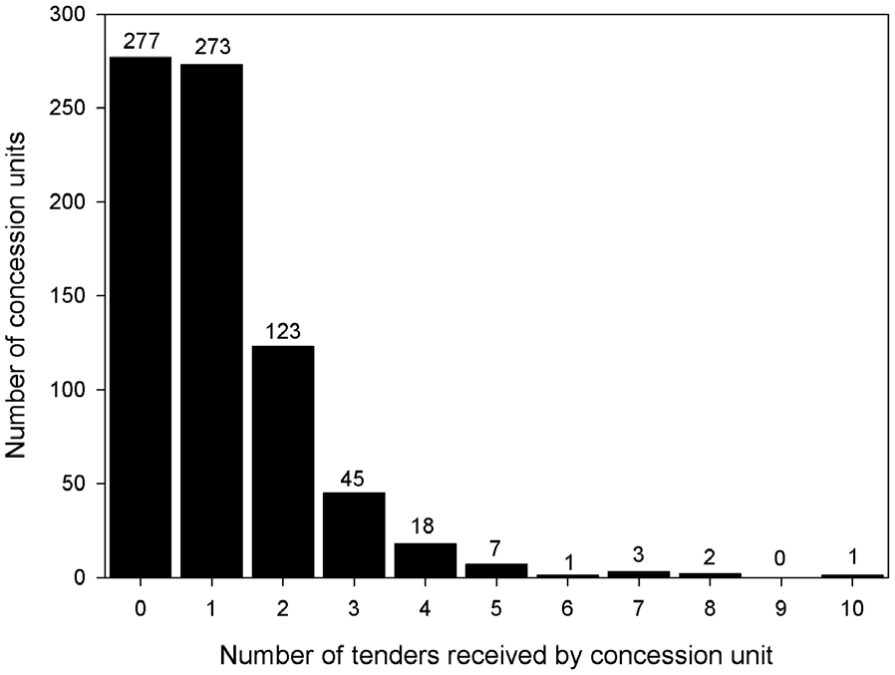
The distribution of the number of tenders received by the concession units (min 0; max 10).

The allocation process revealed that there was more offer than demand for concession units in Loreto; up to 37 per cent (n = 277) of all the units did not receive a tender at all and only 27 per cent (n = 200) of the units were subject to competition between two or more applicants. A mere 4 per cent of the units received tenders from more than 3 applicants, the maximum being 10 tenders per unit (Figures 1 and 2).

According to our analysis, the geographical location and the total area of the concession units had a significant effect on their popularity (Table 3; concession Blocks presented in Figure 1). Units in Block B were the most popular, having on average 1.23 tenders per unit (95% confidence intervals [CIs] = 0.96, 1.59), whereas units in Blocks A and C received on average 0.76 tenders (95% CIs = 0.58, 0.99) and 1.06 (95% CIs = 0.87, 1.29) per unit, respectively. Larger concession units received more tenders than smaller ones (Table 3). That is, large concession units had on average 1.10 tenders (95% CIs = 0.88, 1.37) compared to, an average of 0.91 tenders (95% CIs = 0.74, 1.11) in small units.

**Table 3:** The effect of explanatory variables on the number of tenders received by the concession units.

*Predictor*	*df_num,den_*	*F*	*P*
Distance to the closest city	2, 739	6.15	0.0023
Closest city	1, 739	0.07	0.80
Concession Block	2, 739	10.14	<0.0001
Concession unit area	1, 739	5.12	0.024
Estimated timber volume/ha	1, 739	0.35	0.55
Median skidding distance	1, 739	0.60	0.44

The river distance to the closest city (Iquitos or Pucallpa) was also significantly related to the popularity of a given concession unit (Table 3). Concession units at intermediate distances (500–700 km) from cities received the highest number of tenders (on average 1.28 tenders per unit, 95% CIs = 1.02, 1.62), while the units located closer (on average 0.96 tenders per unit, 95% CIs = 0.79, 1.16) or further from the cities (on average 0.81 tenders per unit 95% CIs = 0.60, 1.07) were less popular. This is consistent with our hypothesis of preference for intermediate distances because of proximity to cities, implying more extraction pressure – and as a consequence less timber left – on the one hand, and growing distance implying more difficult access, on the other. Meanwhile, whether the concession unit was located closer to Iquitos or Pucallpa, estimated timber volumes and median skidding distances of the concession units were not statistically related to the number of tenders they received (Table 3).

### Willingness to pay: area-based fees

**Figure 3:**
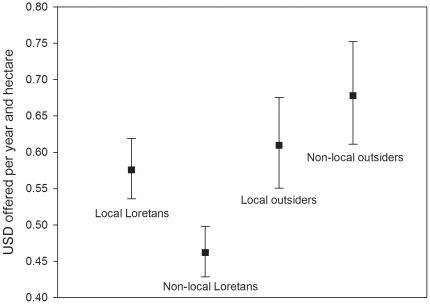
The differences between the offered area-based fees (USD/ha/year) of the four locality groups studied. Squares represent estimated marginal means and error bars their 95% confidence limits.

The highest area-based fee offered was 1.48 USD/ha/year, and the highest winning offer was 1.30 USD/ha/year. The offered area-based fees were related to the locality of the applicants (Table 4). Figure 3 shows that, on average, non-local outsiders offered the highest yearly fees per hectare of concession, contrasting to the non-local Loretans who offered the lowest fees. The economic offers were also related to the scale of actor (Table 4); small extractors offered, on average, lower area-based fees than medium-sized actors (0.52 USD/ha/year [95% CIs = 0.48, 0.57] vs. 0.64 USD/ha/year [95% CIs = 0.60, 0.68], respectively). The type of applicant was also associated with the area-based fees offered (Table 4): partnerships (0.60 USD/ha/year [95% CIs = 0.55, 0.65] and individuals (0.59 USD/ha/year [95% CIs = 0.55, 0.63] made almost equally high offers, whereas companies offered less (0.55 USD/ha/year [95% CIs = 0.50, 0.59].

**Table 4:** The effect of explanatory variables on the area-based fees (USD/ha/year) offered by the applicants.

*Predictor*	*df*	χ^2^	*P*
Locality	3	52.8	<0.0001
Type of actor	2	7.85	0.02
Scale of actor	1	30.0	<0.0001
Closest city	1	6.62	0.01
Concession Block	2	17.2	0.0002
Concession unit area	1	1.69	0.19
Median skidding distance	1	0.41	0.52
Estimated timber volume/ha	1	4.78	0.029
Distance to the closest city	2	8.87	0.012

Concession units in certain Blocks received higher offers than units in other Blocks (Table 4). The units in Block C received the highest offers; an average of 0.65 USD/ha/year (95% CIs = 0.62, 0.72, whereas units in Blocks A and B received on average offers of 0.52 (95% CIs = 0.47, 0.58) and 0.55 USD/ha/year (95% CIs = 0.50, 0.61), respectively. Distance to the closest city also had a significant effect on the area-based fees offered (Table 4); concession units with short (0.60 USD/ha/year [95% CIs = 0.56, 0.64] and intermediate (0.60 USD/ha/year [95% CIs = 0.56, 0.65] distances from the closest city received, on average, higher offers than those located further (0.53 USD/ha/year [95% CIs = 0.48, 0.59]). Whether the closest city was Iquitos or Pucallpa influenced area-based fees as well, since in the case of Iquitos the area-based fees were, on average, lower (0.53 USD/ha/year [95% CIs = 0.51, 0.55]) than in the case of Pucallpa (on average 0.63 USD/ha/year [95% CIs = 0.55, 0.71]). Moreover, higher estimated timber volumes per hectare attracted somewhat higher offers than lower ones (0.60 [95% CIs = 0.57, 0.64] USD/ha/year vs. 0.55 [95% CIs = 0.50, 0.61]). Neither the distance to the river network (median skidding distance) nor the concession unit area were statistically related to the area-based fees offered.

### Winners and losers: success of tenders

**Figure 4:**
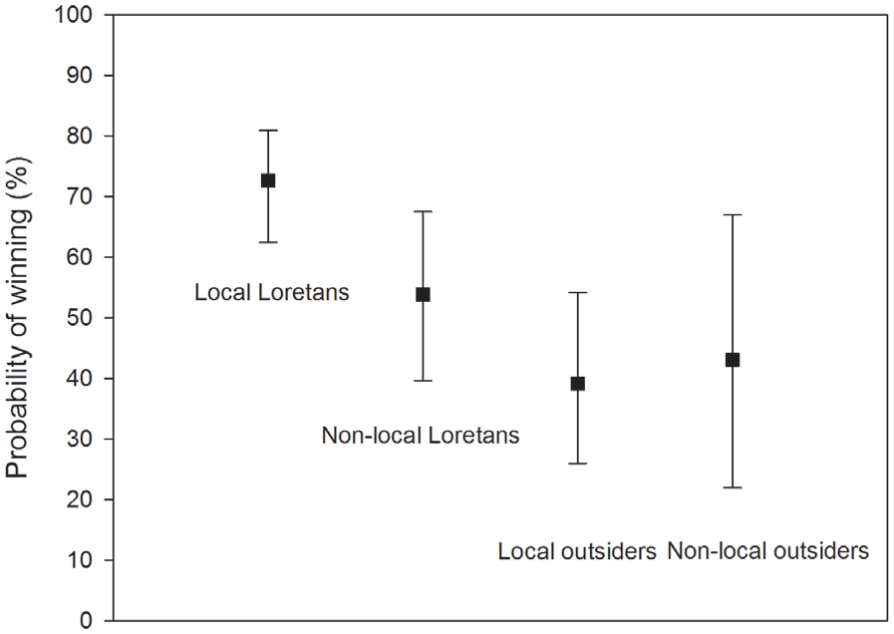
The differences between the probabilities (%) of the applicants for winning a concession unit in the four locality groups studied. Squares represent estimated marginal means and error bars their 95% confidence limits.

Table 5 shows that Local Loretans won more than half (1.56 million ha) of all the concession land that was finally allocated in the tendering (2.58 million ha). There was a statistically significant relationship between the locality and the success of the applicants (Table 6). Local Loretans had the highest probability of winning, compared to non-local Loretans, local outsiders, and non-local outsiders (Figure 4). It thus seems that in addition to applicants having previous ties to the forest areas they solicited, Loretans in general benefitted from the tendering's scoring system. Applicants registered outside Loreto, whether or not they were considered locals, seemed to have lower probabilities of winning a concession.

**Table 5:** The number of concession units and concession area (hectares) applied and won by applicants representing different locality and scale classes.

	Units applied	Units won	Win%	Hectares applied	Hectares won	Win%
**Locality**						
Local Loretans	380	250	66	2.376,520	1.564,015	66
Non-local Loretans	156	89	57	973,099	536,962	55
Local outsiders	100	42	42	668,699	276,101	41
Non-local outsiders	131	32	24	827,845	206,731	25
*Total*	*767*	*413*	*54*	*4.846,163*	*2.583,809*	*53*
**Scale**						
Small	227	126	56	1.483,923	821,621	55
Medium-scale	591	299	51	3.680,719	1.840,677	50
*Total*	*818*	*425*	*52*	*5.164,642*	*2.662,298*	*52*

**Table 6:** The effect of explanatory variables on whether or not the applicant's tender won the race for a concession.

*Predictor*	*df*	χ^2^	*P*
Locality	3	19.55	0.0002
Type of actor	2	0.11	0.95
Scale of actor	1	2.45	0.12
Area-based fee	1	0.37	0.55

Although medium-sized actors won the majority of the concession land (1.84 million ha), a considerable area (0.82 million ha) was also allocated to those small-scale extractors only soliciting one concession unit (Table 5). Neither the area-based fees offered by the applicants, nor the type or scale of actor, were statistically related to the applicants' success (Table 6).

## Discussion

Our analysis revealed three general tendencies in the forest concession allocation process of Loreto. First, there was little true competition in the tendering; second, the area-based fees offered did not significantly affect the results of the tenders; and third, the Loreto-based applicants had a higher probability of winning a concession unit than those based outside Loreto.

In the end, only a quarter of all the offered concession units were subject to two or more competing tenders. However, without the rules of the game clearly favouring the small and the local, the process could arguably have lost its legitimacy before it was even initiated. Previous examples show that it would have also been possible to restrict the participation in the process only to small actors, as was the case in two tenderings organized in the Madre de Dios and Ucayali regions in 2003 [20]. In Loreto, however, the allocation system turned out to emphasise the local experience of the participants rather than their size. Actors registered in Loreto and with local experience in the form of previous logging contracts, or residence near the solicited concession units, achieved the largest share of the units and concession area. This indicates that the allocation method in general, and the scoring system in particular, were successfully designed to serve the purpose of favouring applicants that had already established ties with the offered forest areas.

While this was one way in which the process succeeded in achieving at least some degree of legitimacy within the region, the tendering simultaneously failed to fundamentally change the power-relations regulating access to timber resources in Peruvian Amazonia [4]–[5], [29]–[31]. What was important for the forest industrialists was to guarantee a steady flow of timber, and local small-scale extractors depending on locally organized chains of trade are vital for this kind of supply. What these small-scale extractors needed was to bolster their direct access to the forest. Although the allocation process left large areas of forest in the hands of small actors, it is not certain to which degree things have changed in the field. According to a recent report published by the Environmental Investigation Agency [4], with the concessions in function for several years now, it is still commonplace in Loreto that urban timber merchants equip small-scale extractors by advance payments which frequently feed a circle of debt and impoverishment.

Another anxiety that was frequently voiced before the tendering was that of forest inventories, operative plans, and management planning required by the forest law, proving to be prohibitively costly and thus making concessions not attainable for small extractors [32]. This viewpoint cannot be ruled out as an explanation for the low level of competition in the tendering. Furthermore, the possibility of small and local actors being used only as legal representatives of larger timber merchants or companies cannot be straightforwardly ruled out, but according to our analysis, the fear of large national or foreign capital overwhelming small and local applicants – a common concern before the tendering [33] – proved to be unfounded. While the concession rights are transferable, i.e. the contracts can be further traded, the commitment of local actors can be reinforced through new options based on a wider variety of forest values. New approaches embedded in the future forest regime could contribute positively to the Peruvian efforts to halt forest degradation in and around logging areas. There are several experiences that can be studied to identify such approaches.

### Policy implications

Most Amazonian countries face problems similar to those of Peru, and many have undergone forest regime reforms during the last 15 years. In Ecuador and Colombia, the forest regime is in need of reform, but the lack of detailed analyses of their particular characteristics hinders comparisons to other Amazonian countries' forest sector reforms. Neither of these two countries currently applies long-term forest concessions as a major administrative arrangement. In Colombia, a new forest law decreed in 2006 introduced a system based on forest concessions, but the law was declared inconstitutional and revoked in 2008 because its preparation did not adequately address issues related to consultation of local and indigenous communities [34]. In Ecuador, the forest policy development has recently been described as unpredictable [35]. In Bolivia and Brazil, large scale forest concessions have been implemented, and their reforms have received more attention internationally [35]–[36], but comparative studies between Amazonian countries are still largely lacking.

Recently, major efforts to reshape the forest sector in Peru have been a consequence of international agreements binding the Peruvian government to reform forest legislation while also implementing and enforcing the current rules more efficiently [4], [10], [37]–[38]. Particularly the free trade agreement between Peru and the US has driven changes in the Peruvian legislation [4]. A package of laws related to the free trade agreement, including a new forest law, was approved in 2008. The new law (‘Legislative decree 1090’) included mechanisms aimed at opening Amazonia for new investment, such as forest concessions solicited through private initiative, and it also enabled drastic changes in land-use designations. In Amazonia, and particularly among the region's indigenous population, these changes were commonly seen as intended to facilitate the privatisation of indigenous peoples' traditional lands. Consequently, protests culminating in tragic acts of violence in northern Peru between state agents and indigenous protesters in June 2009 forced the government to revoke the most controversial of these laws, including the forest law [10].

Currently, a draft of a new forest law is being discussed in a process claimed by the government to be more participatory than the previous one [4], [39], yet controversies still remain as to the implementation of the reform, not least regarding the indigenous and local communities' land rights issues and the concessionaires' ability and willingness to follow the law [40]. As a part of any future regime, Peru is in need of new approaches integrating wider environmental values also to the forest concession mechanism. Today, fortunately, there is more diversity than ever of possible additional values that can be directly linked to sustainable forest management. Peruvian forest concessions are intended to be a long-term commitment to forest management; in addition to the timber and non-timber resources that the forests under concession contain, they also entail vital, albeit hard-to-value, ecosystem services [10], [41].

Forest concessions will most likely form an important part of the National Programme of Forest Conservation for Climate Change Mitigation [3], promoted by the Peruvian government. The kind of arrangements that will be applied as a part of the Peruvian programme in areas surrounding the forest concessions will certainly affect their feasibility, and vice versa. The Peruvian programme aims at safeguarding the forest cover in an area of 54 million hectares, representing 75 per cent of Peruvian forests. The programme is also planned to include direct area-based payments to its main beneficiaries for conserving forests in their possession. The beneficiaries are defined as “[…] the entitled native and rural communities and population that lives in and around the tropical Amazonian and dry forests of the country” [3]. It remains to be seen how this will be achieved, and what the role of local small scale forest concessionaires will be in this deal.
